# 5,6-Dihydro-5,6-Epoxymultiplolide A, Cytosporone C, and Uridine Production by *Diaporthe hongkongensis*, an Endophytic Fungus from *Minquartia guianensis*

**DOI:** 10.3390/microorganisms13040792

**Published:** 2025-03-31

**Authors:** Andrei da Silva Alexandre, Luana Lopes Casas, David Ribeiro da Silva, Cecilia Veronica Nunez

**Affiliations:** 1 Bioprospecting and Biotechnology Laboratory, Technology and Innovation Coordination, National Institute of Amazonian Research, Manaus 69067-375, AM, Brazil; dreialexandre@gmail.com (A.d.S.A.); luanacasas05@gmail.com (L.L.C.); david.ribeiro07@gmail.com (D.R.d.S.); 2 Graduate Program in Biotechnology and Natural Resources of the Amazon, School of Health Sciences, Amazonas State University (UEA), Manaus 69050-010, AM, Brazil

**Keywords:** fungus, parboiled rice, secondary metabolites, NMR spectroscopy, multiplolide, polyketide, nucleoside

## Abstract

Endophytic fungi are valuable sources of bioactive secondary metabolites, with potential applications in pharmaceutical and agricultural fields. This study investigates the metabolic potential of *Diaporthe hongkongensis*, an endophytic fungus isolated from *Minquartia guianensis*. To date, no secondary metabolites have been identified from this species, highlighting the novelty of this research and its contribution to understanding the chemical diversity of endophytic fungi. The fungus was cultivated on parboiled rice under static and dark conditions for 28 days, leading to the isolation of the following three compounds: 5,6-dihydro-5,6-epoxymultiplolide A (**1**), cytosporone C (**2**), and uridine (**3**). Structural identification was carried out using nuclear magnetic resonance (NMR) spectroscopy and mass spectrometry. The results revealed the metabolic versatility of *D. hongkongensis*, as demonstrated by its ability to produce structurally diverse substances with biological relevance. Hence, it describes the first isolation of secondary metabolites from the endophytic fungus *D. hongkongensis*, marking a significant step in understanding its chemical profile. The identification of a known antifungal compound and a lactone derivative underscores the biosynthetic potential of this endophytic fungus, while the isolation of a nucleoside expands the chemical repertoire of fungal metabolites, suggesting possible roles in cellular metabolism and stress adaptation. These findings highlight the role of endophytic fungi as prolific sources of structurally diverse and potentially bioactive natural products, supporting further exploration of their biotechnological applications.

## 1. Introduction

Fungi of the genus *Diaporthe* are globally distributed and exhibit diverse lifestyles, including endophytic, pathogenic, and saprophytic roles. Notably, they are prolific producers of secondary metabolites, such as polyketides, terpenoids, and alkaloids, which possess a wide range of biological activities, including cytotoxic, antifungal, antibacterial, antiviral, antioxidant, anti-inflammatory, and phytotoxic effects [1,2,3,4]. Despite extensive research on secondary metabolites from *Diaporthe* species, the biosynthetic potential of *Diaporthe hongkongensis* remains largely unknown. A few previous studies on this endophytic fungus have identified only the chemical classes present in its extracts and their potential antifungal activity [5]. However, no metabolites of this species have been reported to date, and its biosynthetic pathways, ecological interactions, and potential for bioactive compound production remain unexplored.

The Amazon rainforest offers a vast reservoir of biological and chemical diversity. This region harbors countless plant species, many of which maintain complex relationships with endophytic fungi. These symbiotic fungi are considered to be a sustainable and underutilized resource for bioprospecting [6]. Among the Amazonian flora, *Minquartia guianensis* Aubl. (family Coulaceae) is an ecologically and culturally significant tree species [7,8]. This plant is particularly rich in triterpenes and a limited number of phenolic bioactive compounds have been identified, highlighting its potential for medicinal and biotechnological applications [9,10]. Despite its ecological and economic relevance, the microbial endophytes associated with *M. guianensis* remain largely unexplored, leaving a substantial gap in understanding their chemical diversity and ecological functions. As endophytic fungi are known to influence secondary metabolite production in host plants, investigating *D. hongkongensis* in this ecological context may provide valuable insights into its biosynthetic capacity and potential functional interactions [11].

The chemical investigation of endophytic fungi such as *D. hongkongensis* is particularly significant due to their potential to yield bioactive compounds [5]. Recent studies on *Diaporthe* species have identified metabolites with promising antibacterial, antifungal, and anticancer activities, many of which are derived from polyketide and nucleoside biosynthetic pathways [1,12,13,14]. Notably, other studies involving *Diaporthe* species cultivated on a solid rice-based medium have demonstrated enhanced production of secondary metabolites, highlighting the importance of optimized cultivation conditions for maximizing metabolic output [1]. Advances in fungal genomics have further enabled the identification of biosynthetic gene clusters responsible for these metabolites, enhancing our understanding of their chemical diversity and potential applications [11]. However, studies on the biosynthetic pathways of *D. hongkongensis* remain scarce, limiting our ability to predict or harness its full metabolic potential.

In this context, the objectives of this study were to isolate and identify secondary metabolites from *D. hongkongensis* and explore its potential as a source of bioactive compounds utilizing a solid rice-base medium. Given that *M. guianensis* is rich in promising substances, investigating its associated fungal endophytes could reveal novel metabolic interactions and secondary metabolites with pharmaceutical potential. This research seeks to address existing gaps in the knowledge of *D. hongkongensis* by elucidating its biosynthetic capabilities and ecological interactions. Furthermore, it contributes to the broader understanding of fungal bioprospecting by linking the biodiversity of the Amazon to its chemical potential. Ultimately, this research reinforces the importance of preserving Amazonian biodiversity and integrating fungal bioprospecting into strategies for sustainable development.

## 2. Materials and Methods

### 2.1. Fungal Material

The endophytic strain *D. hongkongensis* was isolated from fresh, healthy leaves of *Minquartia guianensis* collected in May 2014 from the Reserva Florestal Adolpho Ducke, Manaus, Amazonas, Brazil, at coordinates 2°55′24.69″ S and 59°58′26.12″ W. The study was registered in the National System for the Management of Genetic Heritage and Associated Traditional Knowledge (SisGen/MMA—BR) under the number ACCD2F4.

The fungus was isolated under sterile conditions from the inner tissue of the leaves, following a previously described isolation protocol [5]. Identification was carried out using molecular biological techniques involving DNA amplification and sequencing of the ITS region. The results showed that the fungus was closely related to *D. hongkongensis*, with a 99% similarity in the ITS sequence. Combined with morphological characteristics, the strain was identified as *D. hongkongensis* (Supplementary Table S1 and Figure S1). The fungal strain was deposited in the culture collection of the Laboratory of Bioprospecting and Biotechnology at the National Institute of Amazonian Research (INPA).

### 2.2. Fermentation, Extraction, and Isolation of Compounds

The strain was cultured on potato dextrose agar (PDA) at 28 °C for five days. Four fragments of the agar containing the fungal growth were then excised and aseptically inoculated into three 500 mL Erlenmeyer flasks, each containing a parboiled rice medium. The rice medium consisted of 50 g rice and 100 mL distilled water sterilized at 120 °C for 15 min prior to use. The flasks were incubated for 28 days at 25 °C under static, light-free conditions. After the incubation period, the three rice cultures were extracted four times with ethyl acetate (EtOAc) using an ultrasonic bath (Unique, Ultra Cleaner, Brazil) for 20 min per flask. Additionally, an uninoculated rice medium was incubated and extracted in the same manner after 28 days, serving as a negative control (Supplementary Figure S2). The resulting extracts were filtered and concentrated using a rotary evaporator at 40 °C. The EtOAc extract obtained from the three cultures was subsequently analyzed using thin-layer chromatography (TLC) and hydrogen nuclear magnetic resonance (^1^H NMR) spectroscopy to characterize the classes of secondary metabolites present [15,16].

The EtOAc extract (5.0 g) was subjected to chromatographic separation using a Sephadex LH-20 column eluted with methanol (100% MeOH). A total of 23 fractions were collected and subsequently grouped based on separation patterns observed on silica TLC analytical plates (Sigma-Aldrich). Fraction DhC2f7-8 (0.630 g) was further purified by column chromatography on silica gel 60 (SiO_2_, 230–400 mesh, h = 33 cm, and ø = 2 cm) using a stepwise gradient of dichloromethane (100% DCM), DCM/EtOAc (9:1, 8:2, 7:3, 6:4, and 1:1), 100% EtOAc, and EtOAc/MeOH (9:1, 8:2, 7:3, and 1:1) followed by 100% MeOH, yielding 200 fractions. Refraction DhC2.6F116-119 (0.009 g) was subjected to column chromatography on SiO_2_ (h = 18 cm; ø = 1 cm) using DCM/EtOAc (8:2, 7:3, 6:4, and 1:1) and 100% EtOAc, yielding compound **1** (0.002 g). Similarly, refraction DhC2.1f94-95 (0.005 g) was purified by column chromatography on SiO_2_ (h = 9 cm; ø = 1 cm) with 100% DCM and DCM/EtOAc (9:1, 8:2, and 7:3), yielding compound **2** (0.004 g). Finally, refraction DhC2.5f192-195 (0.020 g) was chromatographed over a SiO_2_ column (h = 28 cm; ø = 1 cm) using 100% DCM, DCM/MeOH (9:1, 8:2, 7:3, and 1:1), and 100% MeOH, yielding refraction C2.5.1f9-10 (0.008 g). This fraction was further purified using column chromatography on SiO_2_ (h = 15 cm; ø = 1 cm), affording compound **3** (0.002 g).

### 2.3. Structural Identification

The substances were solubilized in deuterated acetone (Cambridge Isotope Laboratories, Tewksbury, MA, USA) and deuterated dimethyl sulfoxide (DMSO-*d*_6_; Cambridge Isotope Laboratories). The nuclear magnetic resonance (NMR) spectra were obtained using a Fourier 300 (Bruker, Billerica, MA, USA) spectrometer at the Analytical Center of the INPA, operating at 300 MHz for ^1^H uni- and bidimensional analyses, such as COSY (correlated spectroscopy) and HSQC (heteronuclear single quantum correlation), and at 75 MHz for ^13^C (broadband decoupling [BBD] and distortionless enhancement by polarization transfer [DEPT] at 135°). Chemical shifts (δ) were expressed in parts per million and coupling constants (*J*) were expressed in Hz to describe peak multiplicities, using tetramethylsilane (TMS) as an internal standard.

Mass spectrometry analyses were performed in both positive and negative ionization modes using an ESI (electrospray ionization) source. LC-DAD-MS analyses (ultra-fast liquid chromatography coupled to a diode array detector and mass spectrometer) were conducted using a Prominence UFLC chromatograph (Shimadzu, Kyoto, Japan), equipped with an LC-20AT binary pump, an SPD-M20A diode array detector, an SIL-20A automatic injector, and a MicroTOF-Q II mass spectrometer (Bruker).

## 3. Results

**Figure 1:**
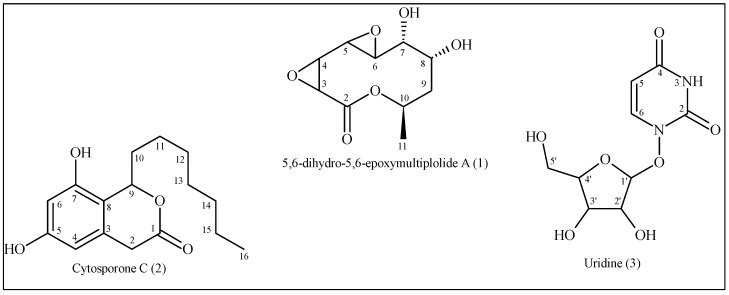
Chemical structure of the substances isolated from the ethyl acetate extract of the fungus *D. hongkongensis*.

The mycelia and culture media of the endophytic fungus *D. hongkongensis* were extracted with EtOAc, yielding a single extract. This extract was concentrated and then repeatedly chromatographed over Sephadex LH-20 and SiO_2_ to yield the following three compounds: a macrolide, 5,6-dihydro-5,6-epoxymultiplolide A (**1**), together with the known cytosporone C (**2**) and uridine (**3**) (Figure 1).

### 3.1. Structural Identification of the Compounds

#### 3.1.1. 5,6-Dihydro-5,6-Epoxymultiplolide A (**1**)

Compound **1** was isolated as a colorless oil. Its molecular formula, C_10_H_14_O_6_, was determined using high-resolution electrospray ionization time-of-flight mass spectrometry (HR-ESI-TOF-MS), which displayed a molecular ion peak at *m*/*z* 253.0680 [M + Na]⁺ (calculated for C_10_H_14_O_6_Na, *m*/*z* 253.0683) (Supplementary Figure S3).

The ^1^H NMR spectrum (300 MHz; acetone-*d*_6_) displayed key signals characteristic of the compound’s structure. The epoxide ring was confirmed by the appearance of two doublets at δ 3.62 and δ 3.64 (*J* = 4.9 Hz), corresponding with geminal hydrogens at C-3 and C-4. A multiplet at δ 3.30 and a double doublet at δ 3.43 (*J* = 2.2; 0.6 Hz) were assigned to the oxygenated methine hydrogens H-5 and H-6, respectively. Other signals included δ 4.07 (multiplet) and δ 4.12 (ddd; *J* = 8.2, 2.2, and 0.9 Hz) for the oxygenated hydrogens H-7 and H-8, as well as δ 1.49 (dd; *J* = 15.5 and 5.0 Hz) and δ 2.29 (ddd; *J* = 15.5, 8.2, and 3.7 Hz) for the H-9 methylene group. The methine hydrogen was observed at δ 5.38 (ddq; *J* = 6.6, 5.0, and 3.7; H-10), and the methyl group at C-11 appeared as a doublet at δ 1.30 (*J* = 6.6 Hz) (Supplementary Figure S4).

The ^13^C NMR spectrum (75 MHz; acetone-*d_6_*) revealed ten carbon signals consistent with the molecular formula. The lactone carbonyl group was identified at δ 166.6 (C-2). Other significant signals included oxygenated sp^3^ carbons at δ 51.2 (C-3), δ 52.8 (C-4), δ 55.9 (C-5), δ 50.3 (C-6), δ 69.8 (C-7), δ 66.9 (C-8), and δ 68.0 (C-10) (Supplementary Figure S5). The methylene carbon (C-9) resonated at δ 35.3, while the methyl carbon (C-11) was observed at δ 17.7 (DEPT 135°, Supplementary Figure S6).

The HSQC spectrum confirmed direct hydrogen–carbon one-bond correlations, facilitating assignments of hydrogens to their corresponding carbons. The epoxide hydrogens at δ 3.62 and δ 3.64 were correlated with δ 51.2 (C-3) and δ 52.8 (C-4), respectively. The oxygenated methine hydrogens δ 3.30 (H-5) and δ 3.43 (H-6) showed correlations with δ 55.9 (C-5) and δ 50.3 (C-6). Similarly, the hydrogen at δ 4.07 (H-7) correlated with δ 69.8 (C-7), and the methyl hydrogens at δ 1.30 (H-11) were linked to δ 17.7 (C-11) (Supplementary Figure S7).

The HMBC spectrum provided critical long-range correlations, confirming the connectivity of the molecule. The epoxide hydrogens (H-3 and H-4) at δ 3.62 and δ 3.64 showed correlations with the lactone carbonyl carbon (C-2; δ 166.6). H-5 (δ 3.30) exhibited correlations with C-3, C-4, and C-7 (δ 51.2, 52.8, and 69.8), while H-6 (δ 3.43) showed correlations with C-4 (δ 52.8) and C-5 (δ 55.9). The methyl hydrogens (H-11; δ 1.30) displayed correlations with the oxygenated methine carbon (C-10; δ 68.0) and methylene carbon δ 35.3 (C-9) (Supplementary Figure S8)

The COSY spectrum further supported the structure by showing H-H coupling relationships. The epoxide hydrogens (H-3 and H-4) were coupled to each other, confirming their proximity. H-6 (δ 3.43) was coupled to H-8 (δ 4.12), while H-8 was coupled to the methylene hydrogens of H-9 (δ 1.49 and δ 2.29). Additionally, sequential couplings were observed between H-10 and H-11 (δ 1.30) (Supplementary Figure S9).

The data were compared with the literature, confirming the chemical structure for compound **1** [17].

#### 3.1.2. Cytosporone C (**2**)

Compound **2** was isolated as a yellow amorphous solid. The molecular formula was determined to be C_16_H_22_O_4_ based on HR-ESI-TOF-MS, which showed an [M + H]⁺ peak at *m*/*z* 279.1605 (calculated for C_16_H_23_O_4_ as *m*/*z* 279.1590) (Supplementary Figure S10).

The ^1^H NMR spectrum (300 MHz; acetone-d_6_) revealed characteristic signals for the aromatic hydrogens at δ 6.34 (H-6) and δ 6.24 (H-4), as well as a deshielded methine hydrogen at δ 5.54 (H-9), indicative of its proximity to oxygenated carbons. Methylene groups were observed at δ 3.79 and 3.43 (H-2) and in the aliphatic region at δ 1.86–1.27. A terminal methyl group resonated as a triplet at δ 0.87 (H-16; *J* = 6.9 Hz) (Supplementary Figure S11).

The HSQC and HMBC spectra supported these observations, with signals consistent with two aromatic carbons (δ 158.1 for C-5; δ 153.7 for C-7), a ketone carbonyl (δ 170.1; C-1), and an oxygenated methine carbon (δ 77.6; C-9), along with aliphatic and methyl carbons.

The HSQC experiment revealed direct one-bond correlations between hydrogens and their attached carbons, facilitating the assignment of δ 6.34 (H-6) to δ 101.0 (C-6), δ 6.24 (H-4) to δ 105.3 (C-4), and δ 5.54 (H-9) to δ 77.6 (C-9). The methylene hydrogens at δ 3.79 and 3.43 (H-2) correlated with δ 34.5 (C-2), while the terminal methyl hydrogens at δ 0.87 (H-16) were linked to δ 13.4 (C-16) (Supplementary Figure S12).

Long-range correlations from the HMBC spectrum provided key insights into the molecular connectivity. The aromatic hydrogen at δ 6.34 (H-6) showed correlations with δ 105.3 (C-4), δ 112.9 (C-8), δ 153.7 (C-7), and δ 158.1 (C-5), while δ 6.24 (H-6) correlated with δ 158.1 (C-5), δ 112.9 (C-8), δ 101.0 (C-6), and δ 34.5 (C-2). The methine hydrogen at δ 5.54 (H-9) exhibited correlations with δ 112.9 (C-8), δ 153.7 (C-7), δ 132.3 (C-3), and δ 170.1 (C-1), confirming its position adjacent to an oxygenated carbon and an aromatic ring. The methylene hydrogens at δ 3.79 and 3.43 (H-2) correlated with the carbonyl carbon at δ 170.1 (C-1) and the aromatic carbon at δ 132.3 (C-3) (Supplementary Figure S13).

The COSY spectrum established spin–spin couplings between adjacent hydrogens. The methine hydrogen at δ 5.54 (H-9) was coupled to the methylene hydrogen at δ 1.80 (H-10), which were, in turn, coupled to δ 1.45. Sequential couplings were observed from H-11 to H-15 (δ 1.27–1.40), terminating with the terminal methyl group (H-16; δ 0.87) (Supplementary Figure S14).

The data were compared with the literature, confirming the chemical structure for compound **2** [18].

#### 3.1.3. Uridine (**3**)

Compound **3** was obtained as a white solid. HR-ESI-TOF-MS revealed a [M + H]^+^ peak at *m/z* 245.0764 (calculated for C_9_H_13_N_2_O_6_ as *m/z* 245.0770), corresponding with a molecular formula of C_9_H_12_N_2_O_6_ (Supplementary Figure S15).

The structural identification of **3** was carried out based on one-dimensional and two-dimensional NMR spectroscopy data. In the ^1^H NMR spectrum (300 MHz; DMSO-*d*_6_) NH hydrogen of the uracil base appeared as a singlet at δ 11.31, which is characteristic of an amidic hydrogen in a pyrimidine system. The H-6 hydrogen was observed at δ 7.87 (d; *J* = 8.0 Hz), showing a characteristic coupling with H-5 at δ 5.63 (d; *J* = 8.0 Hz). This coupling pattern confirmed the expected spin system of the uracil moiety. The H-1′ anomeric hydrogen of the ribose appeared as a doublet at δ 5.76 (d; *J* = 5.4 Hz), which is indicative of a β-glycosidic bond between the ribose and the uracil base. Hydroxyl hydrogens at δ 5.42 (2′-OH), δ 5.16 (3′-OH), and δ 5.16 (5′-OH) suggested the presence of free hydroxyl groups on the ribose ring, confirming the unmodified nature of the sugar moiety. Multiple peaks between δ 4.01 and 3.58 corresponded with the ribose ring hydrogens, including those at C-2′, C-3′, C-4′, and C-5′. These chemical shifts and coupling patterns were consistent with the expected values for a ribofuranose ring in uridine (Supplementary Figure S16).

The HSQC and HMBC spectra supported these observations revealing the presence of ten distinct carbon resonances, corresponding with the uracil and ribose units. The uracil ring carbons were observed at δ 151.1 (C-2) and δ 163.6 (C-4), which are associated with the carbonyl groups of the pyrimidine system. Additionally, the signals at δ 102.2 (C-5) and δ 141.1 (C-6) were consistent with the values for the conjugated uracil ring. The ribose sugar was identified by the anomeric carbon (C-1′) at δ 88.1. The remaining ribose carbons were found at δ 74.0 (C-2′), δ 70.3 (C-3′), δ 85.2 (C-4′), and δ 61.3 (C-5′), further confirming the ribofuranose configuration. The downfield chemical shifts of C-2′, C-3′, and C-5′ indicated the presence of hydroxyl groups at these positions, supporting the unmodified nature of the ribose unit.

The HSQC spectrum allowed the assignment of carbon–hydrogen pairs. The correlation between δ 5.76 (H-1′) and δ 88.1 (C-1′) reinforced the identity of the anomeric center, while the connections between C-2′ (δ 74.0) and its respective hydrogen further established the ribose framework (Supplementary Figure S17).

The HMBC spectrum provided crucial long-range correlations, particularly confirming glycosidic connectivity and the electronic environment of the uracil base. The correlation between H-1′ (δ 5.76) and C-2 (δ 151.1) and C-6 (δ 141.1) confirmed the direct attachment of the ribose to the uracil base. Additionally, the correlation between H-5 (δ 5.63) and C-4 (δ 163.6) and C-6 (δ 141.1) further supported the structure of the pyrimidine system. The hydroxyl hydrogens (2′-OH, 3′-OH, and 5′-OH) exhibited interactions with their respective ribose carbons, reinforcing the integrity of the sugar moiety (Supplementary Figure S18).

Finally, the COSY experiment revealed coupling interactions, confirming the connectivity between the sugar hydrogens. H-1′ (δ 5.76) coupled to H-2′ (multiplet at δ 4.01–3.95) established the glycosidic bond. Additional coupling between H-2′, H-3′, H-4′, and H-5′ confirmed the expected sequential connectivity of the ribose ring, further supporting the assigned structure (Supplementary Figure S19).

The data were compared with the literature, confirming the chemical structure for compound **3** [19].

#### 3.1.4. NMR Measurements

5,6-dihydro-5,6-epoxymultiplolide A (**1**). ^1^H NMR (300 MHz; acetone-*d*_6_): δ 3.62 (d; *J* = 4.9 Hz; H-3), 3.64 (d; *J* = 4.9 Hz; H-4), 3.30 (m; H-5), 3.43 (dd; *J* = 2.2 and 0.6 Hz; H-6), 4.07 (m; H-7), 4.12 (ddd; *J* = 8.2, 2.2, and 0.9 Hz; H-8), 1.49 (dd; *J* = 15.5 and 5.0 Hz; H-9), 2.29 (ddd; *J* = 15.5, 8.2, and 3.7 Hz; H-9), 5.38 (ddq; *J* = 6.6, 5.0, and 3.7 Hz; H-10), and 1.30 (d; *J* = 6.62 Hz; H-11). ^13^C NMR (75 MHz; acetone-*d*_6_): δ 166.6 (C-2), 51.2 (C-3), 52.8 (C-4), 55.9 (C-5), 50.3 (C-6), 69.8 (C-7), 66.9 (C-8), 35.3 (C-9), 68.0 (C-10), and 17.7 (C-11). Molecular formula: C_10_H_14_O_6_ ascertained by HR-ESI-TOF-MS (*m/z* 253.0683; [M + Na]⁺).

Cytosporone C (**2**). ^1^H NMR (300 MHz; acetone-*d*_6_): δ 6.34 (1H; d; *J* = 0.6 Hz; H-6), 6.24 (1H; d; *J* = 0.6 Hz; H-4), 5.54 (1H; dd; *J* = 8.8 and 5.0 Hz; H-9), 3.79 (1H; d; *J* = 19.2 Hz; H-2), 3.43 (1H; d; *J* = 19.2 Hz; H-2), 1.86 (1H; m; H-10), 1.80 (1H; m; H-10), 1.55 (1H; m; H-11), 1.45 (1H; m; H-11), 1.27–1.40 (8H; m; H-12–15), and 0.87 (3H; t; *J* = 6.9 Hz; H-16). ^13^C NMR (75 MHz; acetone-*d*_6_): δ 170.1 (C-1), 158.1 (C-5), 153.7 (C-7), 132.3 (C-3), 112.9 (C-8), 105.3 (C-4), 101.0 (C-6), 77.6 (C-9), 35.6 (C-10), 34.5 (C-2), 31.7 (C-14), 29.0 (C-13), 29.0 (C-12), 25.5 (C-11), 22.4 (C-15), and 13.4 (C-16). Molecular formula: C_16_H_22_O_4_ ascertained by HR-ESI-TOF-MS (*m/z* 279.1605; [M + H]⁺).

Uridine (**3**). ^1^H NMR (300 MHz; DMSO-*d*_6_): δ 11.31 (1H; H-3), 7.87 (1H; d; *J* = 8.0 Hz; H-6), 5.76 (1H; d; *J* = 5.4 Hz; H-1′), 5.63 (1H; d; *J* = 8.0 Hz; H-5), 5.42 (1H; 2′-OH), 5.16 (1H; 3′-OH), 5.16 (1H; 5′-OH), 4.01 (1H; m; H-2′), 3.95 (1H; m), 3.83 (1H; m; H-4′), and 3.58 (2H; t; *J* = 11 Hz; H-5′). ^13^C NMR (75 MHz; DMSO-*d*_6_): δ 151.1 (C-2), 163.6 (C-4), 102.2 (C-5), 141.1 (C-6), 88.1 (C-1′), 74.0 (C-2′), 70.3 (C-3′), 85.2 (C-4′), and 61.3 (C-5′). Molecular formula: C_9_H_12_N_2_O_6_ ascertained by HR-ESI-TOF-MS (*m/z* 245.0770; [M + H]^+^).

## 4. Discussion

The isolation of 5,6-dihydro-5,6-epoxymultiplolide A (**1**), cytosporone C (**2**), and uridine (**3**) from the endophytic fungus *D. hongkongensis* underlines the importance of optimizing cultivation conditions to stimulate secondary metabolite production. *D. hongkongensis* was cultured on parboiled rice under static conditions at 25 °C in the dark for 28 days. This methodological approach was crucial for inducing the production of secondary metabolites, suggesting that nutrient availability, environmental stress, and cultivation parameters play an integral role in the biosynthetic activity of endophytes [15,20].

The use of parboiled rice as a cultivation medium is particularly noteworthy. Parboiled rice is a carbon- and nitrogen-rich substrate that supports fungal growth while providing a complex matrix that mimics the nutrient heterogeneity of the natural host environment. This substrate has been used in natural product research to induce secondary metabolite production as it creates a solid matrix with low water activity, a condition known to upregulate secondary metabolism in fungi [15,21,22]. The static cultivation mode, combined with prolonged incubation in the dark, likely mimics the low-oxygen and light-deprived environments fungi experience in their natural endophytic niche, further triggering the expression of the biosynthetic gene clusters responsible for metabolite production [20].

Alternative fermentation techniques, such as co-cultivation strategies or liquid-state fermentation, could differentially influence metabolite yield and diversity, potentially leading to the enhanced production of bioactive compounds. However, it was observed that *D. hongkongensis*, as well as other *Diaporthe* species, cultivated in liquid-state fermentation did not produce a diverse array of secondary metabolites, suggesting that solid-state fermentation (SSF) may be more effective for this species [3,5,23,24]. This limitation may be attributed to the homogeneous nature of submerged fermentation, which can sometimes be insufficient to activate the biosynthetic gene clusters involved in secondary metabolite production in filamentous fungi [25]. In contrast, SSF provides a heterogeneous microenvironment that resembles the natural endophytic habitat, leading to a greater diversity of secondary metabolites due to localized gradients in nutrient availability, oxygen diffusion, and water activity. SSF is an alternative to submerged fermentation to produce value-added products [26,27]. The use of parboiled rice in this study likely played a key role in stimulating biosynthesis as its complex composition could act as a metabolic signal to activate secondary metabolism [28]. It is also strongly tied to the concept of waste-to-wealth by valorizing biomass and reducing downstream liquid volume treatment [29].

The isolation of 5,6-dihydro-5,6-epoxymultiplolide A (**1**) represents a significant contribution to the expanding multiploidy family of metabolites. This macrolide compound, previously identified only once in *Phomopsis* sp. (the asexual state of *Diaporthe*), underscores its rarity and the necessity for further research into its biosynthesis and functional role [17]. Structurally, it possesses an epoxide moiety and a lactone ring, features that are commonly associated with potent bioactivity, including cytotoxic, antifungal, and antihyperlipidemic properties [30,31]. The presence of an epoxide group is particularly notable as it enhances the chemical reactivity of the molecule, potentially increasing its interaction with biological targets such as enzymes, cellular receptors, or lipid membranes [32]. Many epoxide-containing natural products have demonstrated antimicrobial and immunomodulatory effects, suggesting that 5,6-dihydro-5,6-epoxymultiplolide A exhibits structural features commonly associated with bioactivity, warranting further investigation into its pharmacological potential. Given the structural resemblance of this compound to other macrolides, its potential applications in antifungal or antibacterial drug development warrant further investigation. Furthermore, the biosynthesis of macrolides such as **1** is typically governed by modular polyketide synthase (PKS) pathways, which are highly regulated by environmental and genetic factors [33,34]. The ability of *D. hongkongensis* to produce this rare compound under solid-state fermentation conditions suggests that specific culture parameters play a crucial role in activating silent biosynthetic gene clusters (BGCs). Such gene clusters are often dormant under standard laboratory conditions but can be induced through modifications to nutrient compositions, stress factors, or co-cultivation approaches [20,35]. Recent advancements in genome mining and transcriptomic analysis have enabled the identification of cryptic biosynthetic pathways in fungi, paving the way for novel metabolite discoveries [36]. The elucidation of the genetic mechanisms controlling the production of 5,6-dihydro-5,6-epoxymultiplolide A could facilitate targeted metabolic engineering strategies to enhance its yield and structural diversity.

Cytosporone C (**2**), another polyketide isolated in this study, has previously been reported in other *Diaporthe* species, indicating a conserved biosynthetic pathway within this genus. Cytosporone C has demonstrated its antifungal activity against the pathogenic fungus *Alternaria solani*, suggesting a potential ecological role in *D. hongkongensis* by inhibiting the growth of competing microorganisms within the plant host [37]. Endophytic fungi frequently establish symbiotic or mutualistic relationships with their hosts by producing bioactive secondary metabolites that suppress pathogenic fungi, modulate microbial communities, or enhance plant defense responses [38]. The production of **2** may contribute to this ecological role by acting as a chemical mediator within the plant–fungal interaction network. In an agricultural context, metabolites such as cytosporone C could be explored for their potential as natural fungicides, offering an alternative to synthetic chemical pesticides that pose risks of environmental persistence and pathogen resistance. Furthermore, cytosporone derivatives have shown bioactivity relevant to human health, including antimicrobial, antimalarial, cytotoxic, antivirus, anti-inflammatory, and allelopathic activity [39,40,41].

Uridine (**3**), a nucleoside essential for nucleotide biosynthesis, was also isolated from *D. hongkongensis*. Uridine plays fundamental roles in cellular metabolism, RNA synthesis, and the regulation of glucose and lipid homeostasis [42]. Beyond its primary metabolic functions, the regenerative effects of uridine in peripheral nerve injuries have been demonstrated in a rat model, highlighting its potential role in nerve repair and regeneration [43]. Its neuroprotective effects have been explored for cognitive enhancement and Alzheimer’s disease treatment, particularly in formulations combining uridine, choline, and DHA [44]. Moreover, uridine has been shown to modulate pyrimidine metabolism, influencing the synthesis of DNA and RNA and acting as a precursor for the biosynthesis of uridine diphosphate sugars (UDP-sugars), which are crucial for glycosylation processes in cells [45]. Uridine’s role in modulating neuroinflammation and its involvement in mitochondrial function further highlight its therapeutic relevance, particularly in neurological disorders [46]. Additionally, uridine analogs are widely utilized in antiviral, anticancer, and antibiotic drug development, targeting essential nucleic acid metabolic pathways in pathogens and tumor cells [47,48]. For instance, certain uridine analogs serve as inhibitors of viral RNA polymerases, making them valuable candidates for antiviral drug development against RNA viruses [49,50]. The biosynthesis of uridine by *D. hongkongensis* suggests potential biotechnological applications, particularly in industrial fermentation processes for nucleoside production. Microbial fermentation methods have been increasingly explored as sustainable and scalable approaches for the synthesis of nucleosides and their derivatives, offering an alternative to chemically intensive synthetic routes [51,52]. The ability of endophytic fungi to naturally produce uridine could be harnessed for pharmaceutical applications, particularly in the large-scale production of therapeutic nucleosides for antiviral and anticancer treatments. Future studies should focus on optimizing fermentation parameters and employing metabolic engineering strategies to enhance uridine yield and structural modifications for improved bioactivity.

From an ecological and functional perspective, the production of 5,6-dihydro-5,6-epoxymultiplolide A, cytosporone C, and uridine by *D. hongkongensis* likely reflects adaptive biochemical strategies that enhance fungal survival and interactions within its host environment. Secondary metabolites frequently function as chemical mediators in microbial interactions, influencing competition, communication, and symbiosis. Many endophytic fungi employ secondary metabolites to outcompete other microbes, protect their plant host from pathogens, or facilitate host colonization [53,54]. Macrolides and polyketides such as **1** and **2** may act as antifungal agents that suppress competing microorganisms, thus providing *D. hongkongensis* with a selective advantage as an endophyte [55,56]. Meanwhile, compound **3** may contribute to stress adaptation and cellular homeostasis, supporting fungal viability under fluctuating environmental conditions [57,58]. The biosynthetic capacity of *D. hongkongensis* suggests a dynamic interplay between endophyte and host, potentially influenced by evolutionary pressures to produce structurally diverse bioactive compounds [59,60]. Given the pharmaceutical and biotechnological relevance of these metabolites, further studies on the biosynthetic pathways and regulatory mechanisms governing their production could provide valuable insights for natural product discovery and metabolic engineering applications [61,62].

## 5. Conclusions

This study provides the first report on the isolation and identification of 5,6-dihydro-5,6-epoxymultiplolide A, cytosporone C, and uridine from *Diaporthe hongkongensis*, highlighting its metabolic versatility and ability to produce structurally diverse secondary metabolites. The successful isolation of these compounds underscores the effectiveness of targeted cultivation strategies, particularly the use of a parboiled rice medium, which proved to be a favorable substrate for stimulating metabolite production. Beyond their chemical diversity, these metabolites suggest ecological and physiological roles that warrant further investigation, particularly in microbial interactions and host adaptation. Their potential applications span multiple fields: in biotechnology, optimizing culture conditions could enhance secondary metabolite production; in pharmaceuticals, 5,6-dihydro-5,6-epoxymultiplolide A and cytosporone C may serve as antimicrobial or cytotoxic agents, while uridine holds promise for antiviral or neuroprotective therapies; in agriculture, cytosporone C could contribute to plant protection by inhibiting pathogenic fungi. Future bioactivity assays and genomic studies will be essential to fully explore their applications in drug discovery and sustainable biotechnological innovations with broad scientific and industrial relevance.

## Data Availability

The data presented in this study are available on request from the corresponding author. The data are part of an ongoing study.

## Supplementary Materials

The following supporting information can be downloaded at: https://www.mdpi.com/article/10.3390/microorganisms13040792/s1. Table S1: Identification result of the fungus *D. hongkongensis* based on GenBank; Figure S1: Phylogenetic tree showing phylogenetic distance among fungi, based on the 18S rRNA gene; Figure S2: EtOAc extract of *D. hongkongensis* and negative control spectra comparison; Figure S3: ESI-MS data of compound 1; Figure S4: ^1^H NMR spectrum of compound 1; Figure S5: ^13^C spectrum of compound 1; Figure S6: DEPT 135° of compound 1; Figure S7: HSQC spectrum of compound 1; Figure S8: HMBC spectrum of compound 1; Figure S9: COSY spectrum of compound 1; Figure S10: ESI-MS data of compound 2; Figure S11: ^1^H NMR spectrum of compound 2; Figure S12: HSQC spectrum of compound 2; Figure S13: HMBC spectrum of compound 2; Figure S14: COSY spectrum of compound 2; Figure S15: ESI-MS data of compound 3; Figure S16: ^1^H NMR spectrum of compound 3; Figure S17: HSQC spectrum of compound 3; Figure S18: HMBC spectrum of compound 3; Figure S19: COSY spectrum of compound 3.
