# Supplementary material for: 5,6-Dihydro-5,6-Epoxymultiplolide A, Cytosporone C, and Uridine Production by Diaporthe hongkongensis, an Endophytic Fungus from Minquartia guianensis
Source: Microorganisms. 2025 Mar 31;13(4):792. doi: 10.3390/microorganisms13040792 (PMC12029568; doi:10.3390/microorganisms13040792)
Supplement: Supplementary file 1 [file microorganisms-13-00792-s001.zip › microorganisms-3500808-supplementary.pdf]

Supplementary Material

Diaporthe hongkongensis Identification

The identification of the fungus was performed by the Coleção de Culturas Tropical Fundação André Tosello (São Paulo, Brazil) and was carried out using molecular biological techniques involving DNA amplification and sequencing of the ITS region. The results showed that the fungus was closely related to *D. hongkongensis*, with a 99% similarity in the ITS sequence. Combined with morphological characteristics, the strain was identified as *D. hongkongensis*.

The DNA was extracted using a commercial kit (UltraClean® Microbial DNA Isolation Kit - MoBio) following the manufacturer's instructions. The target DNA was amplified via Polymerase Chain Reaction (PCR) using the primer oligonucleotides SR6R (5' AAGTATAAGTCGTAACAAGG 3') and LR1 (5' GGTTGGTTTCTTTCCT 3') (White et al., 1990). After electrophoretic separation on a 1.5% (w/v) TBE-agarose gel, the product was purified using the UltraClean® PCR Clean-Up Kit (MoBio). Following another electrophoretic run on a 1.5% (w/v) TBE-agarose gel, the purified product was quantified, concentrated, aliquoted, and supplemented with the oligonucleotide (SR6R) used for sequencing. The sequencing was performed on the ABI 3500 Genetic Analyzer platform (Life Technologies).

The taxonomic classification of the sample named INPA-SB3 was based on sequence comparisons between those obtained from the samples (individually) and those deposited in GenBank (www.ncbi.nlm.nih.gov/) via BLASTn. The sequences were aligned with similar sequences available in the database, and a phenetic tree was constructed using the MEGA6 software. The sample INPA-SB3 exhibited >99% similarity when compared to the most closely matching sequence deposited in GenBank. Therefore, based on the information in this database, the analyzed strain is taxonomically classified as *Diaporthe hongkongensis*, as shown in Table 1 and Figure 1.

Table S1 - Identification result of the fungus *D. hongkongensis* based on GenBank

**Serviço N°**	**Código de Seqüenciamento**	**Resultado**
		**Classificação Taxonômica (NCBI)**
150069-2	**INPA-SB3**	*Diaporthe hongkongensis*
		Eukaryota; Fungi; Dikarya; Ascomycota; Pezizomycotina; Sordariomycetes; Sordariomycetidae; Diaporthales; Diaporthaceae; Diaporthe.

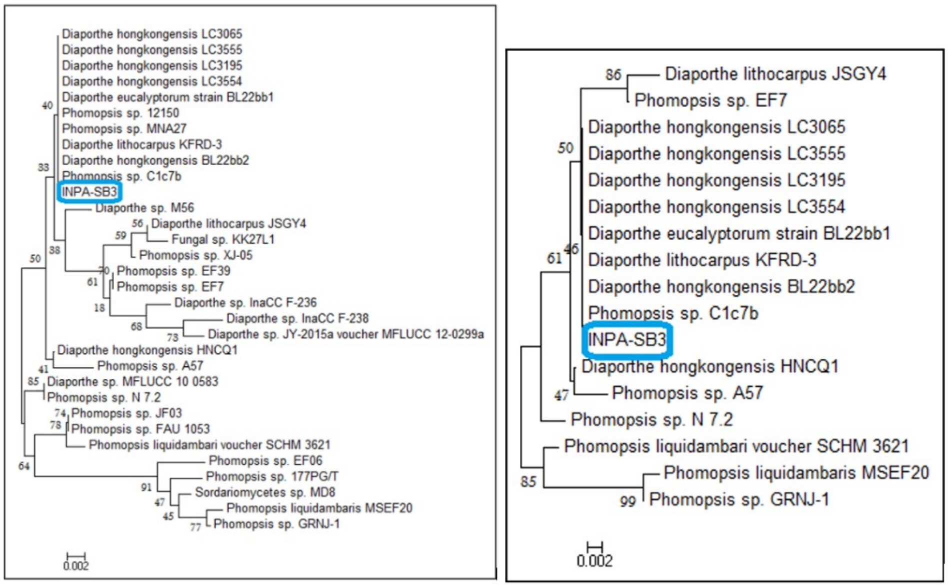

Figure S1 - Phylogenetic tree showing phylogenetic distance among fungi, based on the 18s rRNA gene. Constructed using the MEGA 6.0 software with the Neighbor-Joining method and Tamura-3 parameter, with 1000 Bootstrap replicates

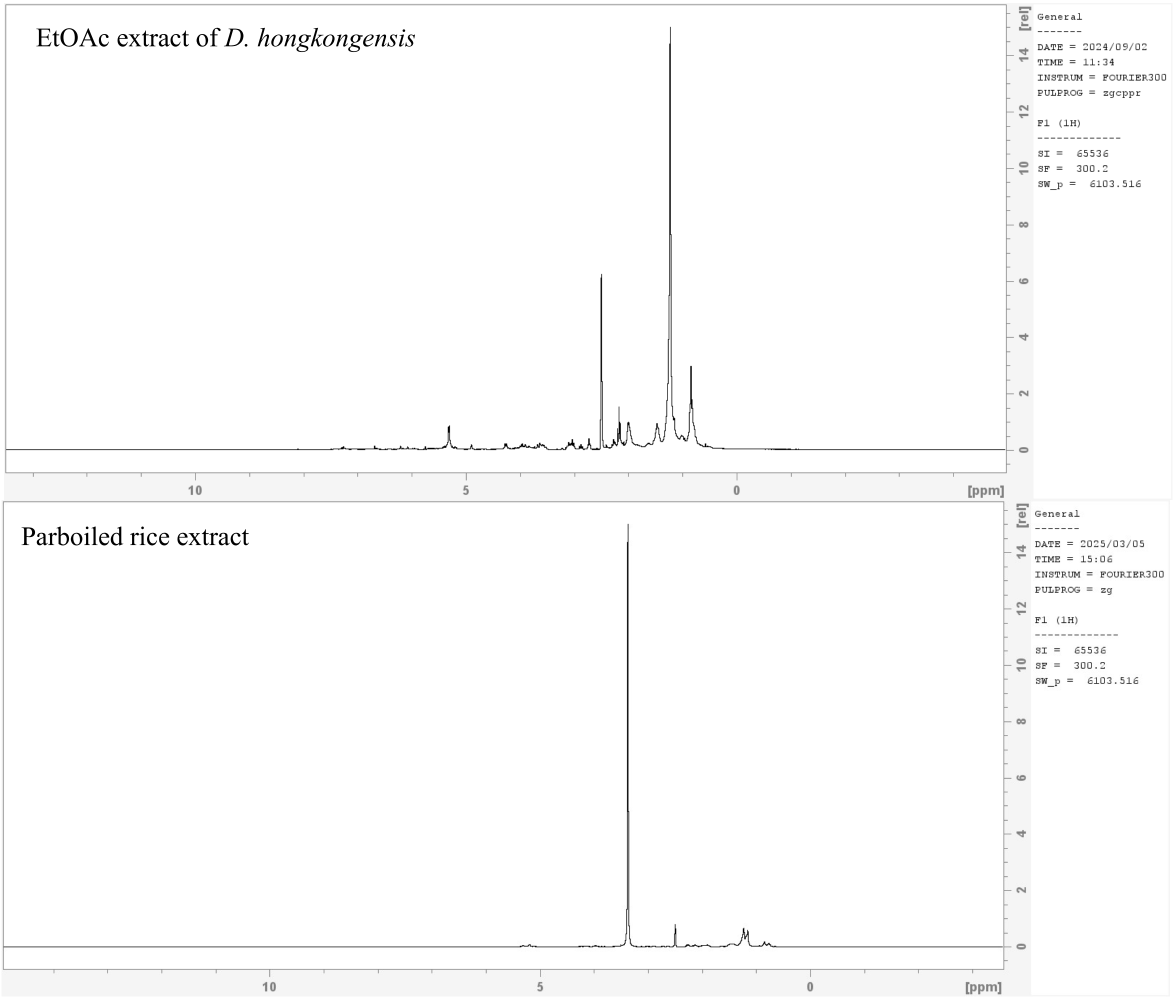

Figure S2 - EtOAc extract of *D. hongkongensis* and negative control (parboiled rice extract) spectra comparison (DMSO-d_6, 300 MHz)

ESI-MS OF COMPOUND 1

HR-ESI-TOF-MS (Positive mode)

MS: *m/z* 253.0680 $[M + H]^+$

Generic Display Report

Analysis Info

Acquisition Date 11/21/2024 1:15:17 PM

Analysis Name D:\Data\Usuarios\2024\Cecilia\Data\Andrei\21-11-2024\ESI+MS2_C26F11-15_20-100_30m_1-2_01_5679.d

Method LC-MS2_ESI+_50-1200_30mi_PosEduardon.m

Operator BDAL@DE

Sample Name ESI+MS2_C26F11-15_20-100_30m

Instrument micrOTOF-Q

Comment Kinetex 2.6u C18 150x2,1mm
Flx=0,2 mL/min - SSplit; P=3001psi; C=1mg/mL(MeOH); Inj=2uL
A(H2O 0,1% ac)/B(MeOH)
0-24 min_20-100%
24-26min_100%
26-28min_100-20%
28-30min_20%
Injetor: MeOH
Calib.:Formiato de Na 10mM_end

Meas. m/z	Adduct	Ion Formula	m/z	err [ppm]	mSigma	Score
213.0754	M+H	C10H13O5	213.0757	1.7	5.0	100.00
231.0863	M+H	C10H15O6	231.0863	0.1	2.8	100.00
253.0680	M+Na	C10H14NaO6	253.0683	1.0	0.9	100.00
461.1648	2M+H	C20H29O12	461.1654	1.3	5.8	100.00
483.1473	2M+Na	C20H28NaO12	483.1473	-0.1	5.4	100.00

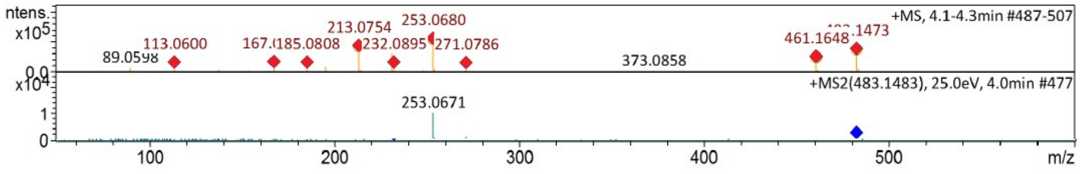

Figure S3 – ESI-MS data of compound 1

SPECTRA OF COMPOUND 1

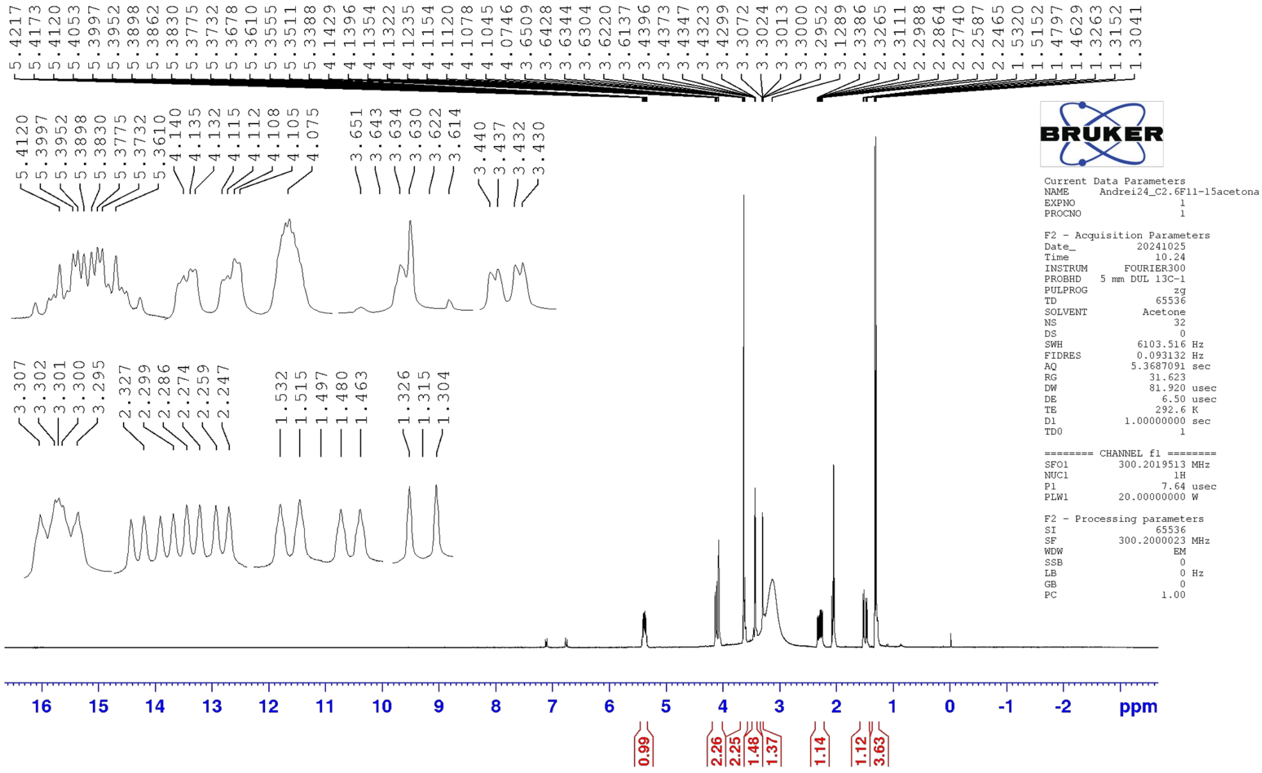

Figure S4 – ^{1}H NMR spectrum of compound 1

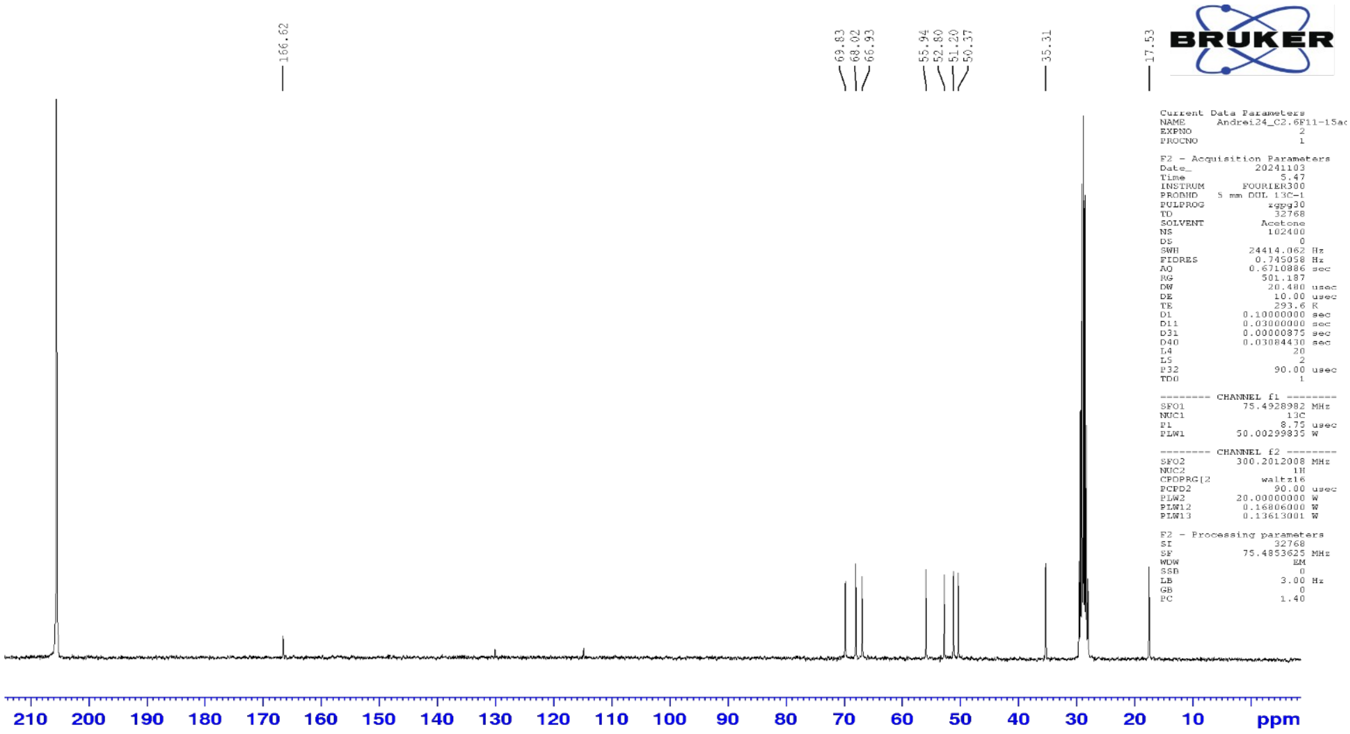

Figure S5 – ^{13}C spectrum of compound 1

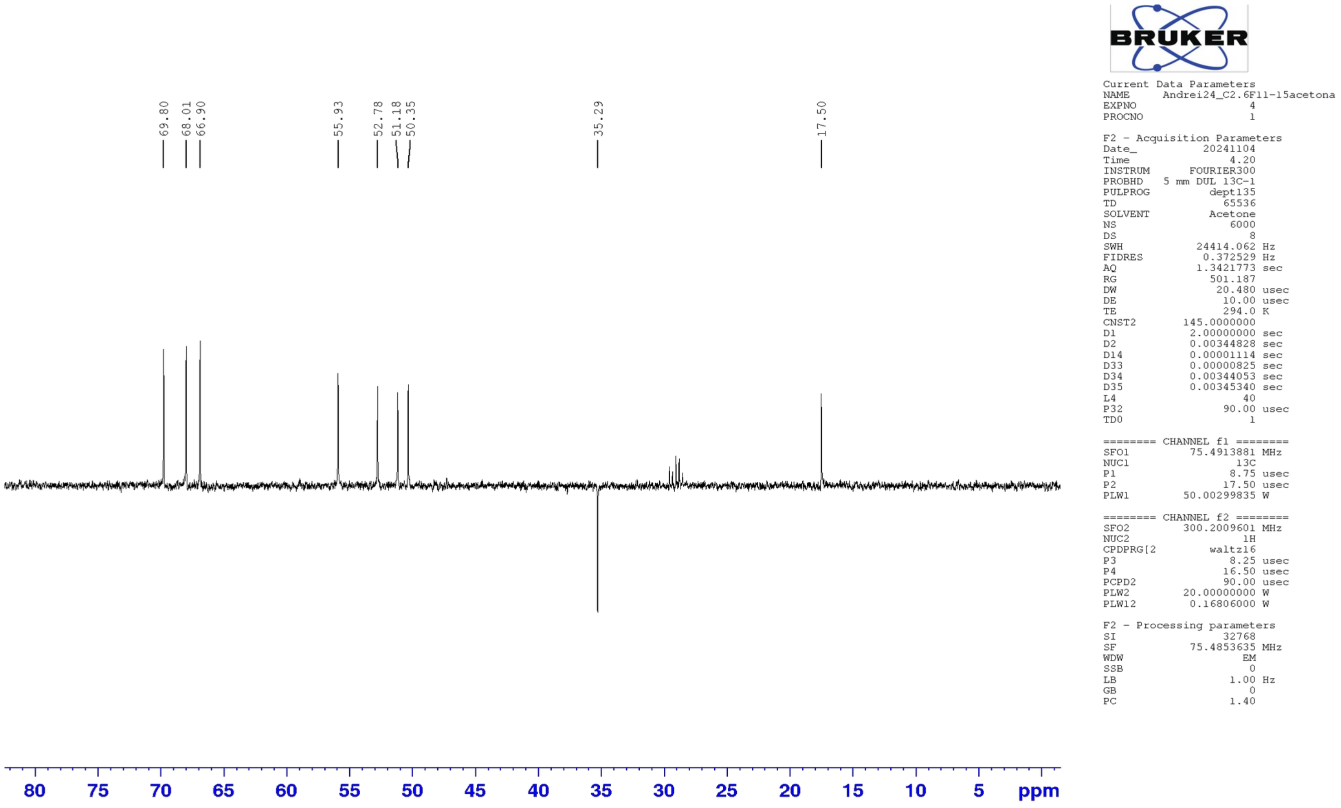

Figure S6 – DEPT 135º spectrum of compound 1

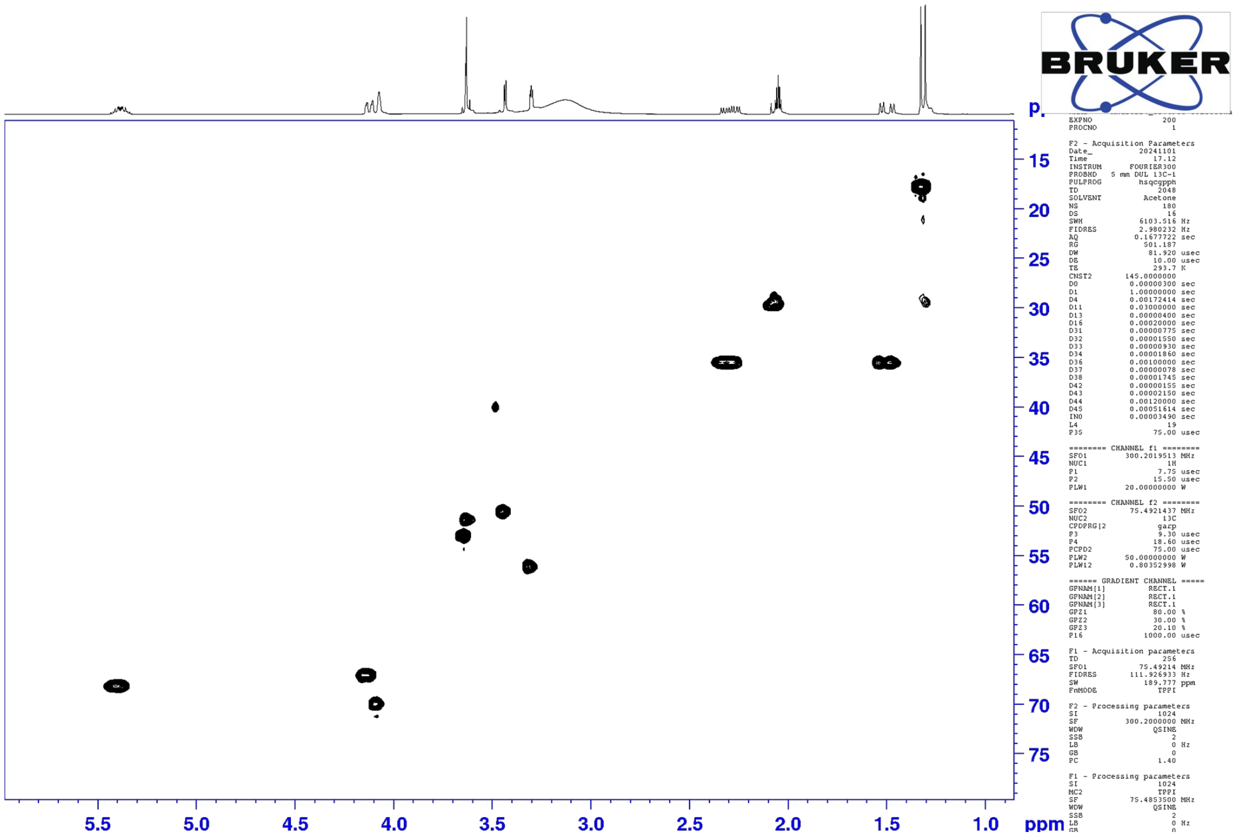

Figure S7 – HSQC spectrum of compound 1

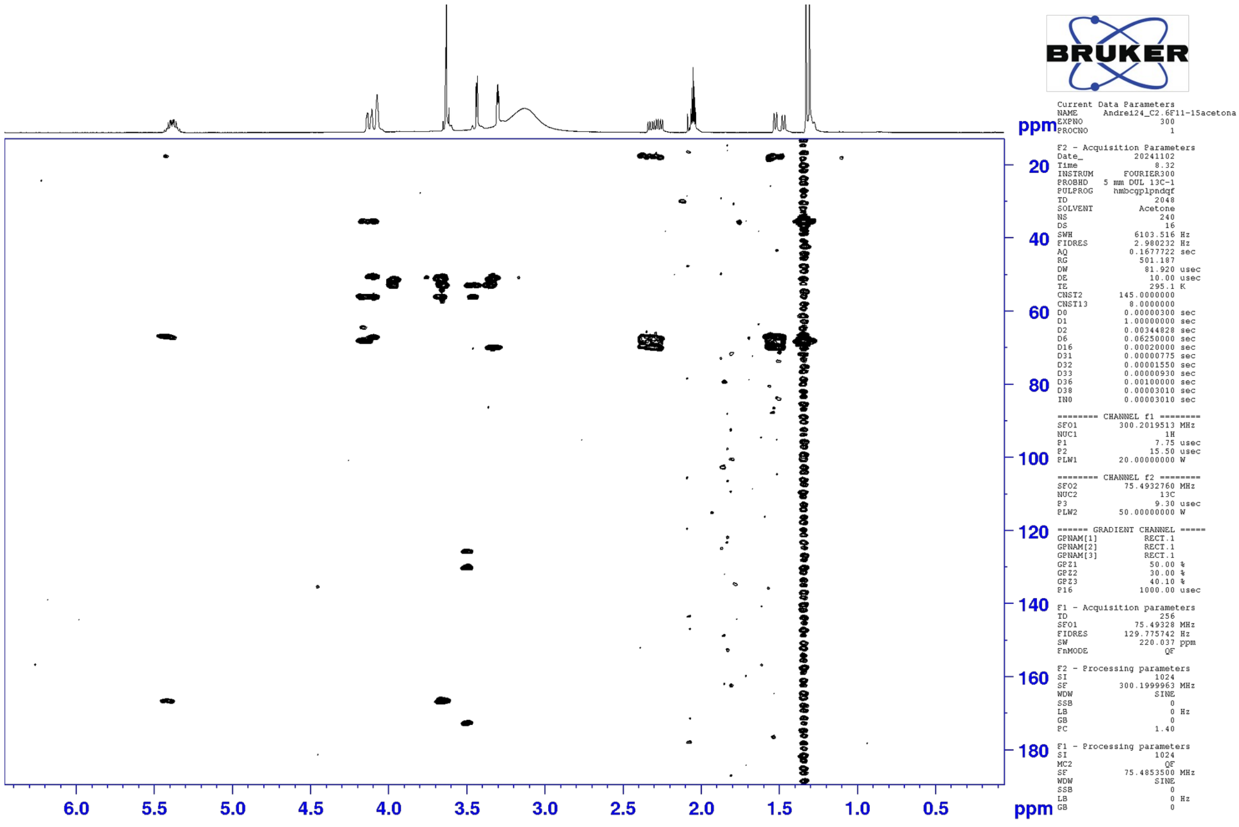

Figure S8 – HMBC spectrum of compound 1

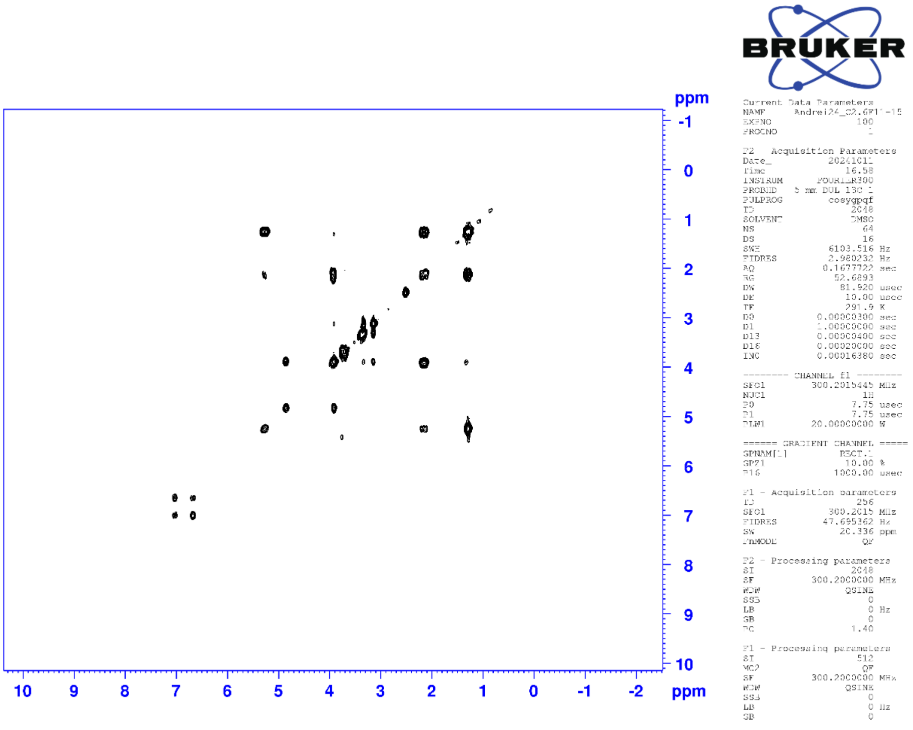

Figure S9 – COSY spectrum of compound 1

ESI-MS OF COMPOUND 2

HR-ESI-TOF-MS (Positive mode)

MS: *m/z* 279.1605 $[M + H]^+$

Generic Display Report (all)

Analysis Info

Acquisition Date 10/30/2024 10:20:44 AM

Analysis Name D:\Data\Usuarios\2024\Cecilia\Data\Andrei\30-10-2024\ESI+MS2_DhC21F2-3_10-100_30m_1-2_01_5610.d

Method LC-MS2_ESI+_50-1200_30mi_PosEduardon.m

Operator BDAL@DE

Sample Name ESI+MS2_DhC21F2-3_10-100_30m

Instrument micrOTOF-Q 228888.10431

Comment Kinetex 2.6u C18 150x2,1mm
Flx=0,2 mL/min - SSplit; P=3230psi; C=1mg/mL(MeOH); Inj=1uL
A(H2O 0,1% ac)/B(MeOH)
0-24 min_10-100%
24-26min_100%
26-28min_100-10%
28-30min_10%
Injetor: MeOH
Calib.:Formiato de Na 10mM_end

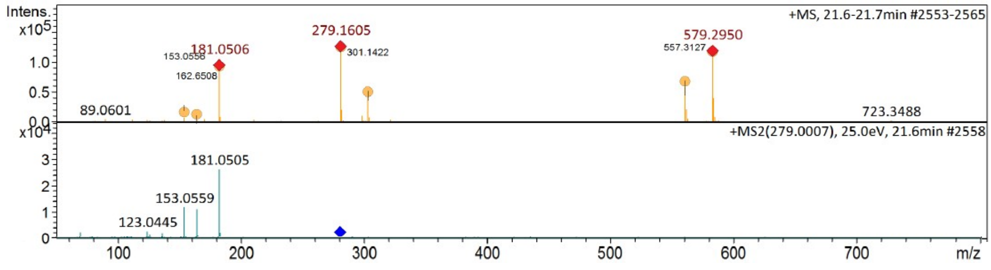

Figure S10 – ESI-MS data of compound 2

SPECTRA OF COMPOUND 2

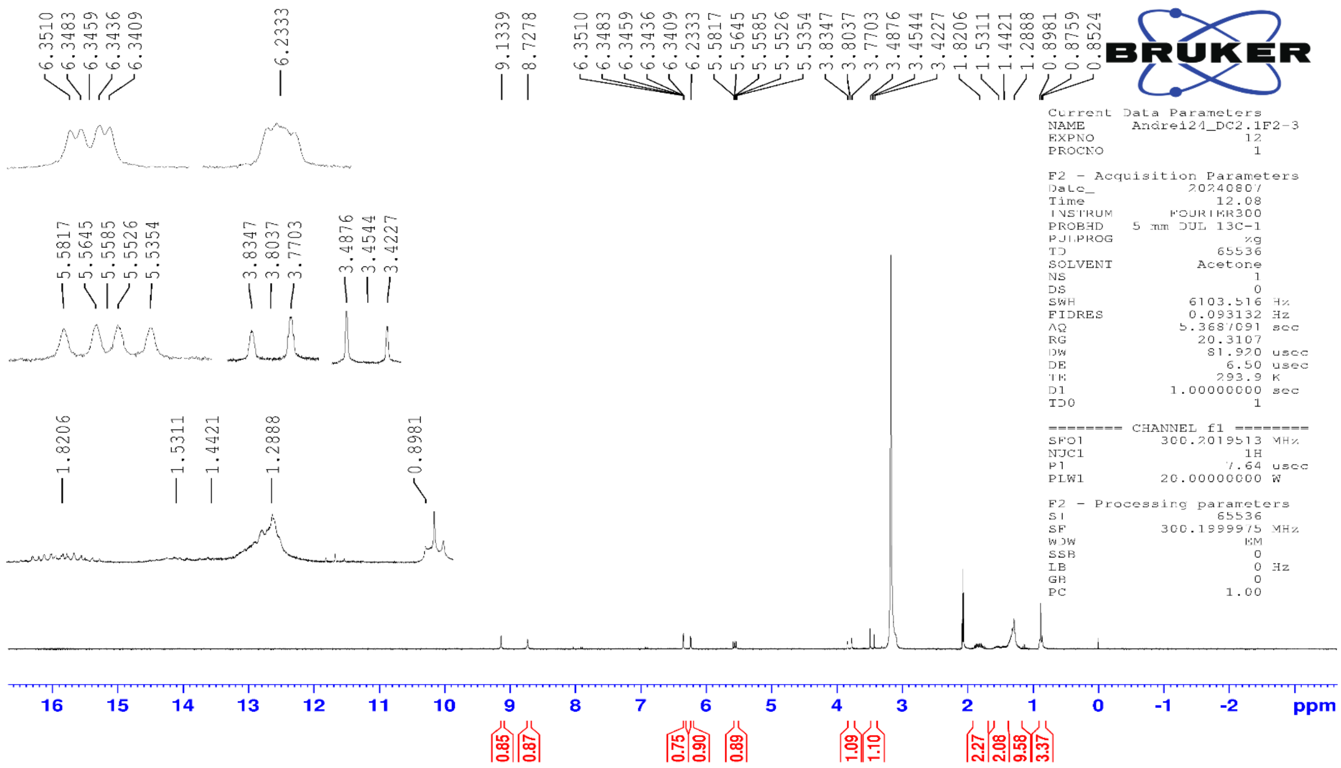

Figure S11 – ^{1}H NMR spectrum of compound 2

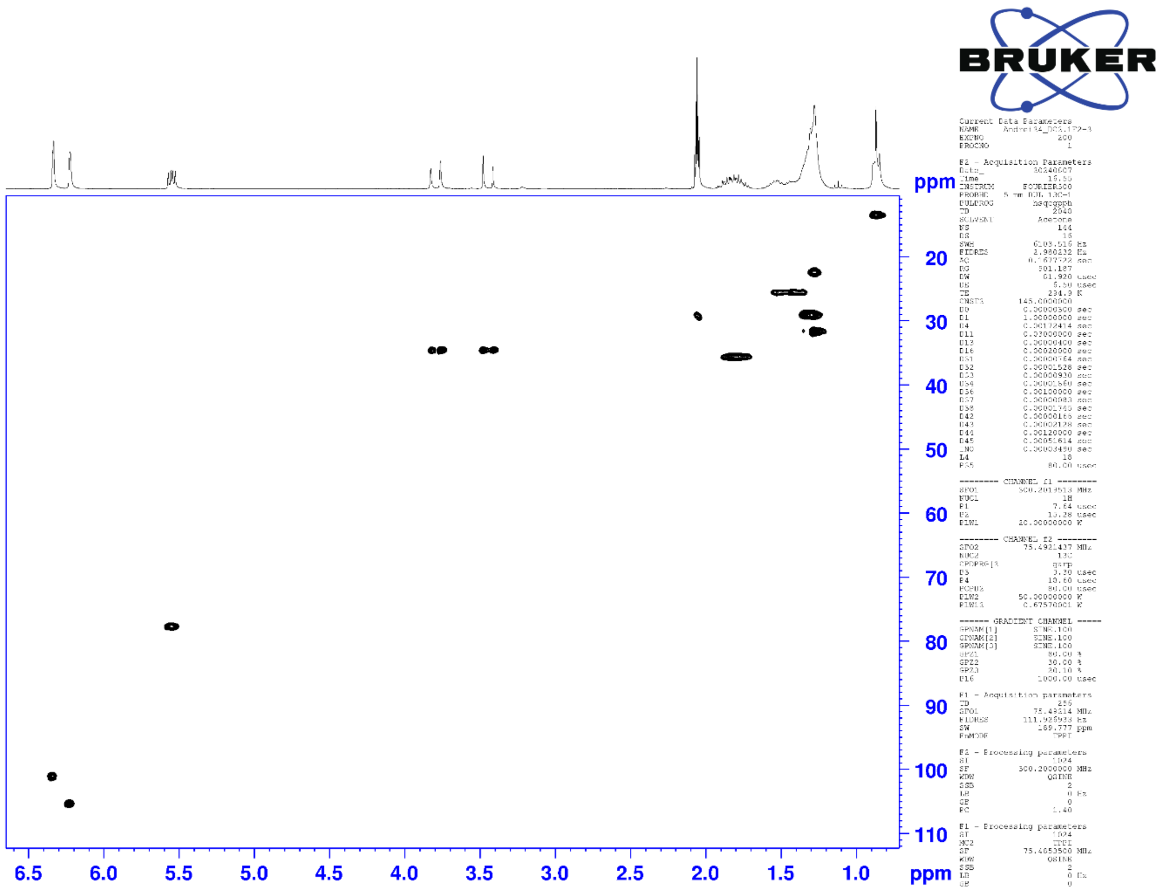

Figure S12 – HSQC spectrum of compound 2

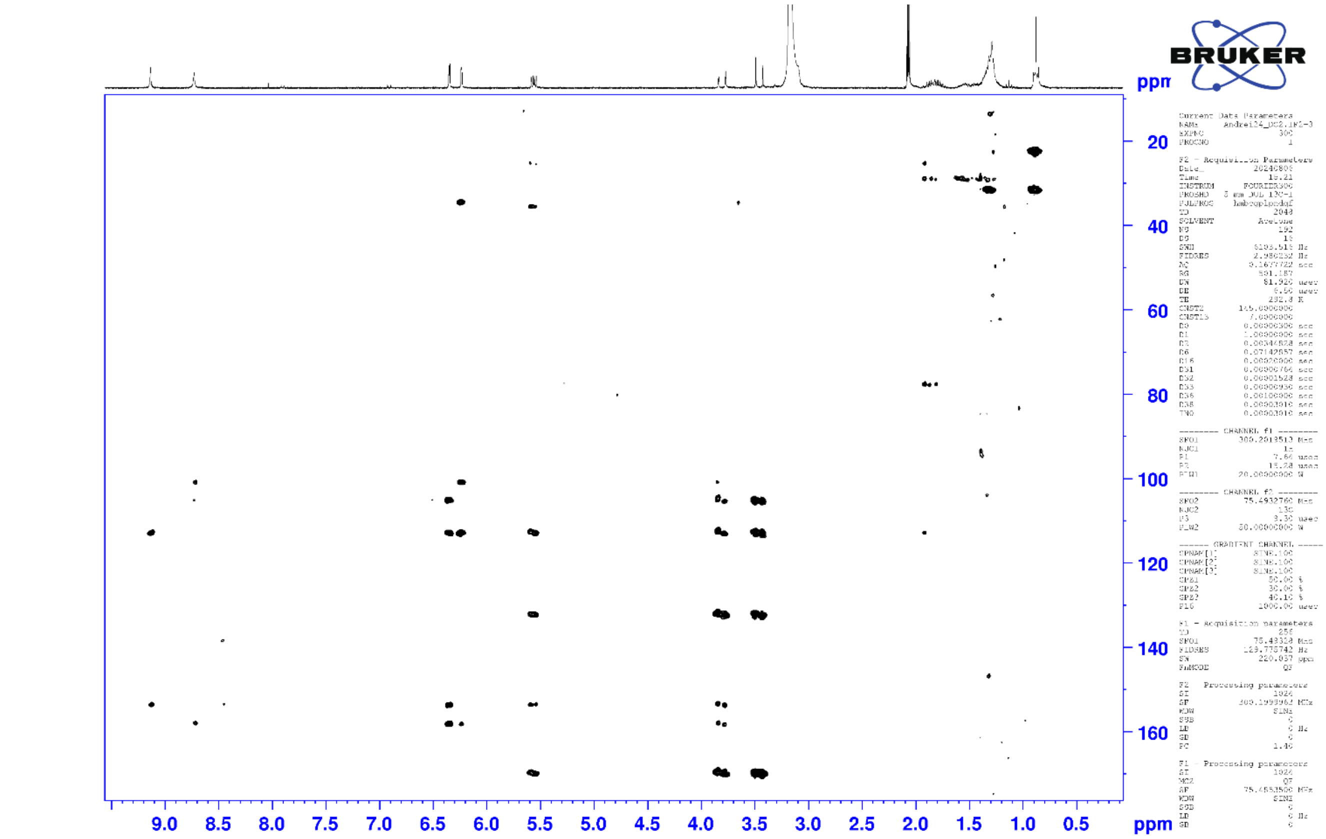

Figure S13 – HMBC spectrum of compound 2

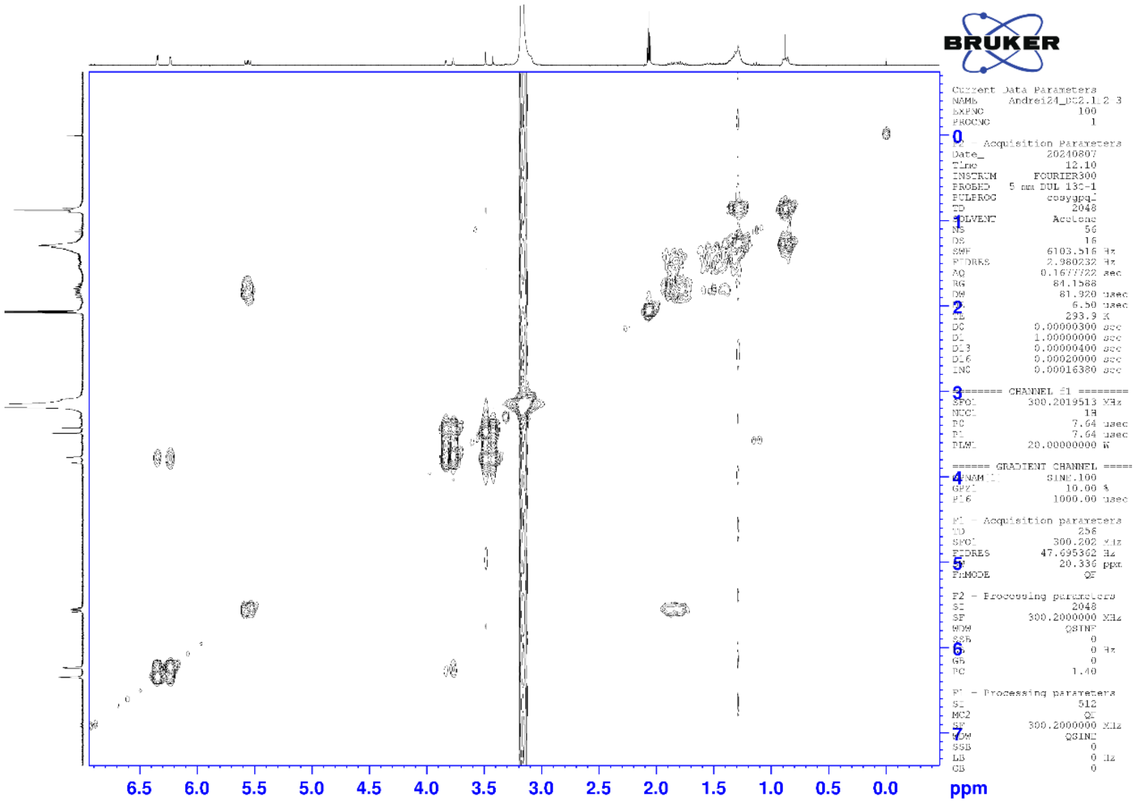

Figure S14 – COSY spectrum of compound 2

ESI-MS OF COMPOUND 3

HR-ESI-TOF-MS (Positive mode)

MS: *m/z* 245.0764 [M + H]$^+$

Generic Display Report (all)

Analysis Info

Analysis Name	D:\Data\Usuarios\2025\Cecilia\Data\Andrei\LC-MS\ESI+_MS2_C2_5-1F3-5_5-25_20m_1-52_01_5776.d	Acquisition Date	1/31/2025 4:03:31 PM
Method	LC-MS2_ESI+_50-1200_20mi_PosEduardon.m	Operator	BDAL@DE
Sample Name	ESI+_MS2_C2_5-1F3-5_5-25_20m	Instrument	micrOTOF-Q 228888.10431
Comment	Kinetex 2.6u C18 150x2,1mmH23-332001<br>Flx=0,2 mL/minSSplit;P=2813psi;C=1mg/mL(MeOH+100uLH2O+3uLAF);Inj=3uL<br>A(H2O 0,1% ac)/B(MeOH)<br>0-3 min_5%<br>3-12min_5-25%<br>12-14min_25-100%<br>14-16min_100%<br>16-18min_100-5%<br>18-20_5%<br>Injetor:MeOH<br>Calib:FormiatoNa10mM_end		

Meas. m/z	#	Ion Formula	m/z	err [ppm]	mSigma	# mSigma	Score	rdb	e⁻ Conf	N-Rule
113.0358	1	C4H5N2O2	113.0346	-11.4	6.7	1	100.00	3.5	even	ok
	2	C9H5	113.0386	24.2	22.7	2	23.88	7.5	even	ok
169.1108	1	C7H13N4O	169.1084	-14.4	12.2	1	100.00	3.5	even	ok
245.0764	1	C9H13N2O6	245.0768	1.5	7.2	1	100.00	4.5	even	ok
	2	C10H9N6O2	245.0781	6.9	8.0	2	45.48	9.5	even	ok
	3	C6H5N12	245.0755	-4.0	10.8	3	68.30	10.5	even	ok

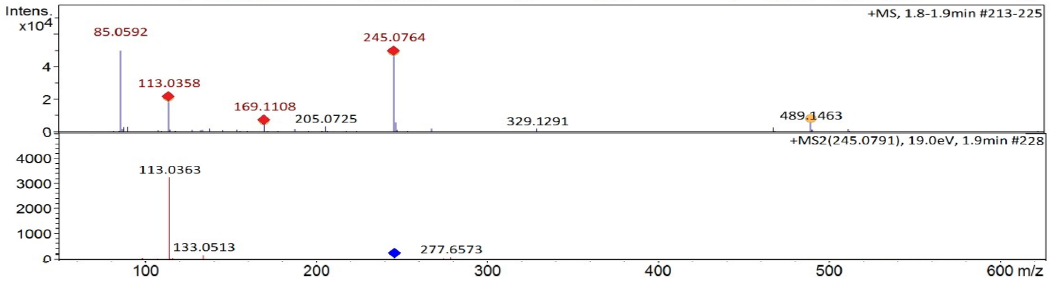

Figure S15 – ESI-MS data of compound 3

SPECTRA OF COMPOUND 3

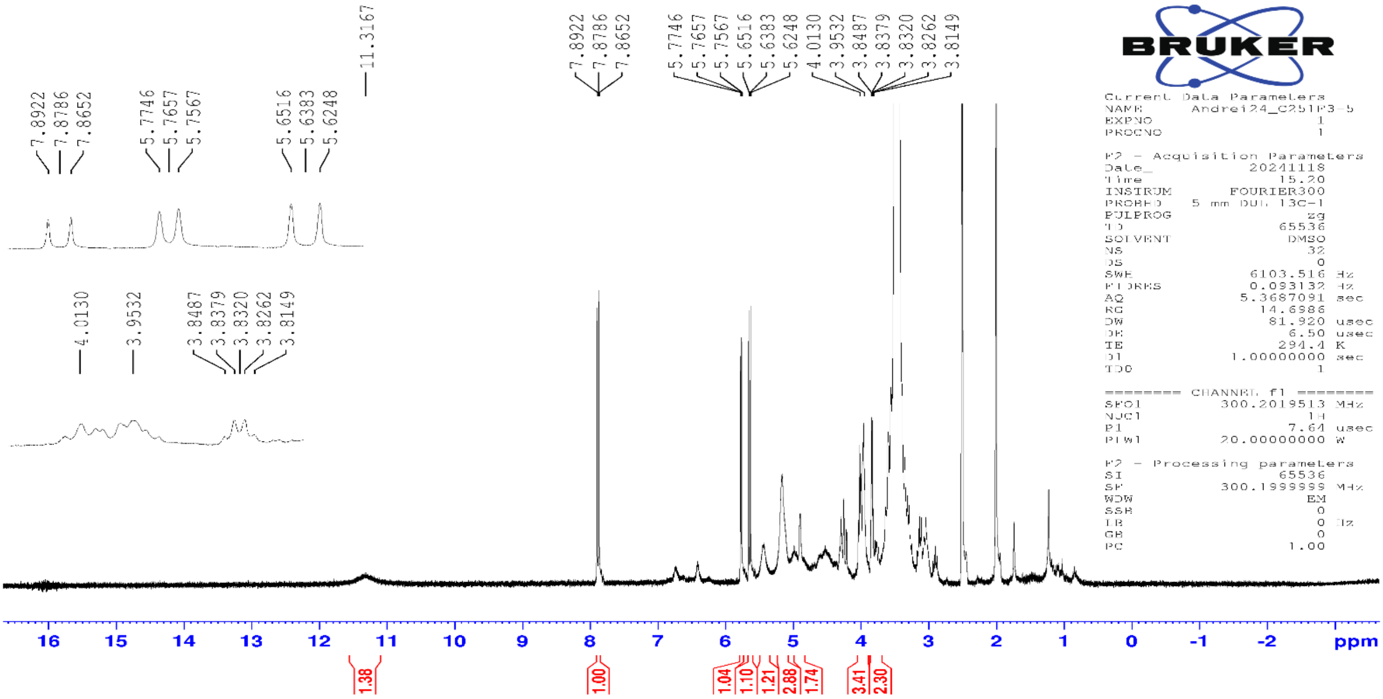

Figure S16 – ^{1}H NMR spectrum of compound 3

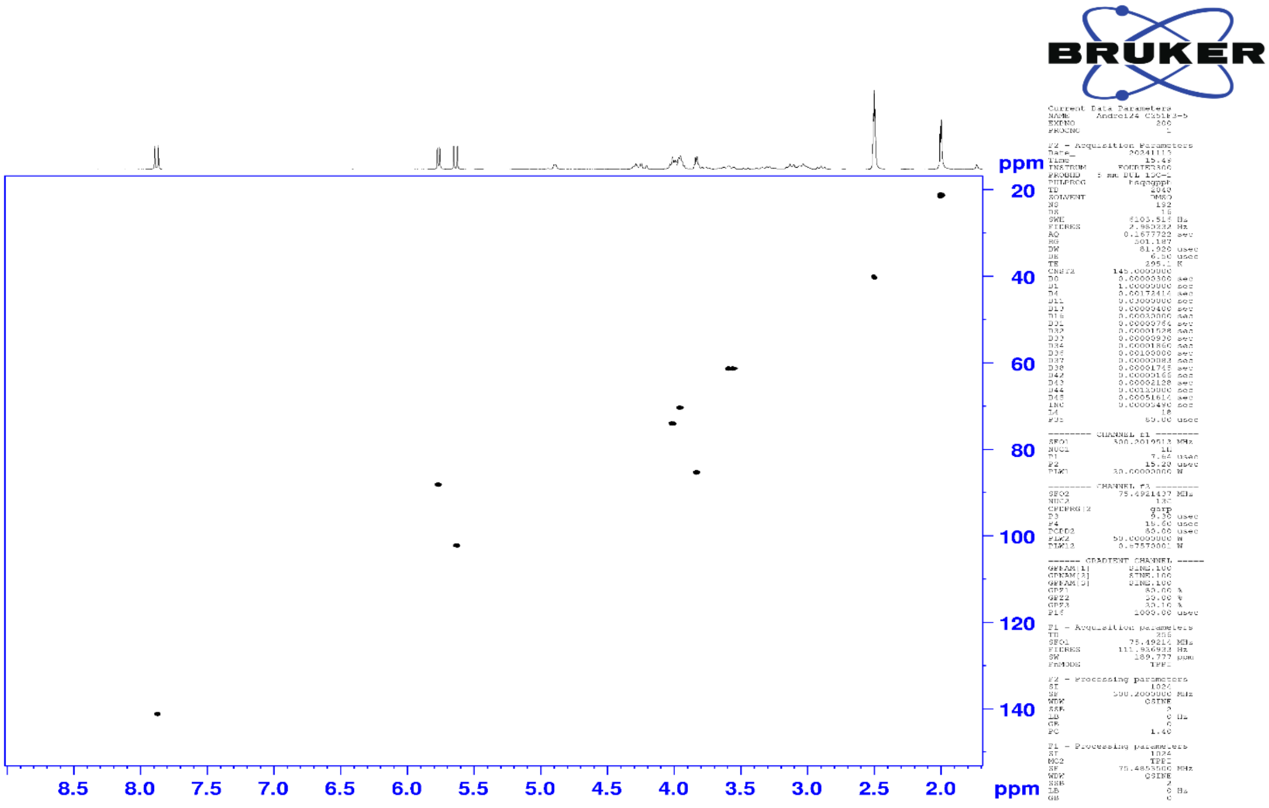

Figure S17 – HSQC spectrum of compound 3

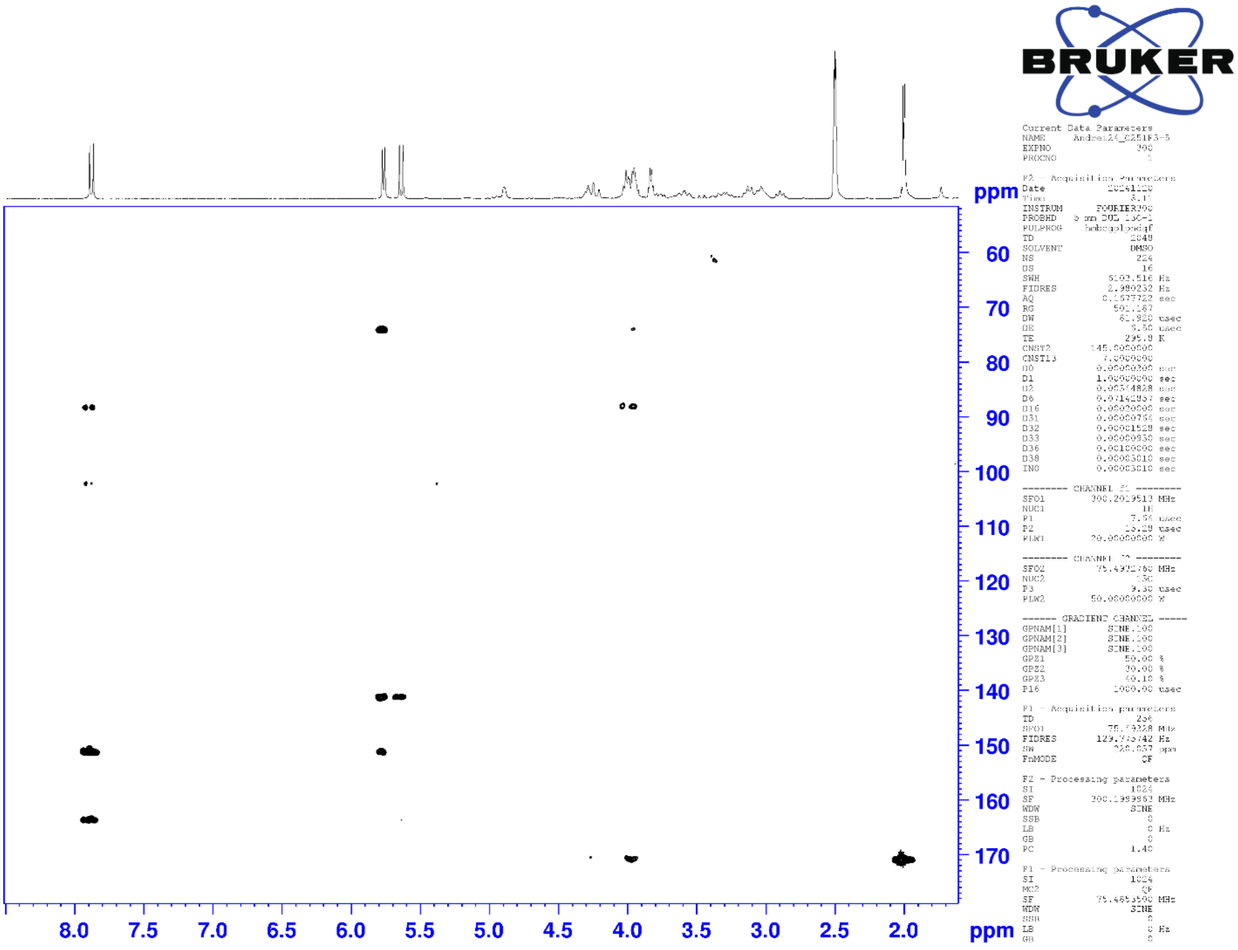

Figure S18 – HMBC spectrum of compound 3

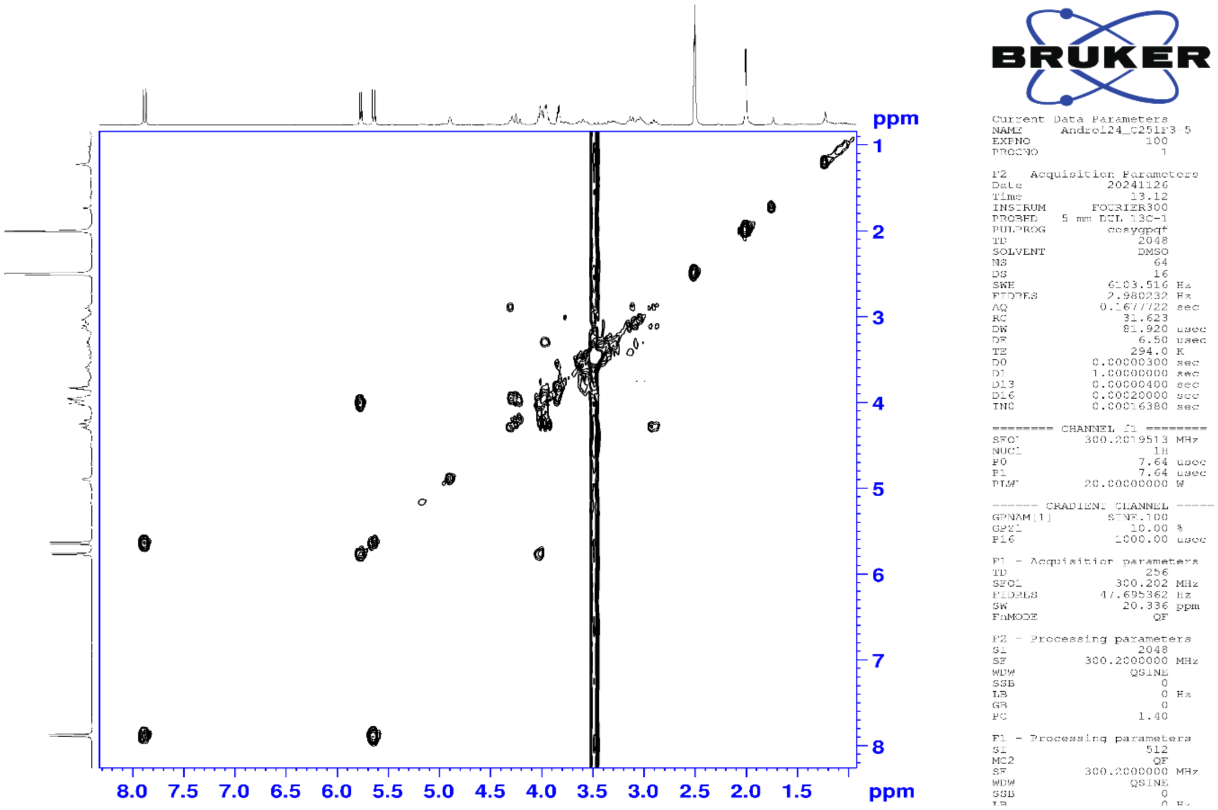

Figure S19 – COSY spectrum of compound 3
